# Most abundant metabolites in tissues of freshwater fish pike-perch (*Sander lucioperca*)

**DOI:** 10.1038/s41598-020-73895-3

**Published:** 2020-10-13

**Authors:** Yuri P. Tsentalovich, Ekaterina A. Zelentsova, Lyudmila V. Yanshole, Vadim V. Yanshole, Iliya M. Odud

**Affiliations:** 1 International Tomography Center SB RAS, Institutskaya 3a, Novosibirsk, 630090 Russia; 2 Novosibirsk State University, Pirogova 2, Novosibirsk, 630090 Russia

**Keywords:** Biochemistry, Ecology, Molecular biology, Zoology

## Abstract

Quantitative metabolomic analysis was performed for eleven tissues of freshwater fish pike-perch (*Sander lucioperca*), including gill, heart, liver, kidney, spleen, muscle, brain, milt, lens, aqueous (AH) and vitreous (VH) humors with the use of NMR spectroscopy. The absolute values of concentrations were determined for more than 65 most abundant metabolites in every tissue. It was found that from the metabolomic viewpoint, kidney and gill are the most similar tissues, while the metabolomic compositions of ocular tissues—lens, AH, and VH significantly differ from that of other tissues. The combinations of intracellular osmolytes and antioxidants are specific for every tissue. In particular, the concentration of antioxidant ovothiol A in the lens is much higher than in any other tissue, while the brain enjoys the elevated level of ascorbate. The most abundant osmolyte in the fish spleen, muscle, and heart is taurine, and in the brain, gill, and lens—*myo*-inositol. Other important osmolytes specific for particular tissues are *N*-acetyl-histidine, *N*-acetyl-aspartate, betaine, threonine-phosphoethanolamine, and serine-phosphoethanolamine. The quantitative data obtained in the present work can be used as the baseline metabolite concentrations in the fish tissues to evaluate the influence of seasonal, ecological and other factors on the fish metabolism.

## Introduction

Metabolomics is the youngest branch of “-omics” sciences emerged at the beginning of twenty-first century. The complete set of small-molecular-weight compounds in tissue, i.e. its metabolome, includes hundreds of metabolites and reflects the actual metabolic processes occurring in the tissue. Pathological processes cause significant changes in the tissue metabolomic composition; therefore, metabolomic analysis is considered as one of the most promising approaches for the early diagnosis and monitoring of a wide spectrum of diseases^1–5^. For that reason, the metabolomes of many human tissues are studied in details^6–12^, including both quantitative metabolomic profiling and changes induced by various diseases. However, animal tissues are studied to a lesser extent, and the metabolomic composition is known for very limited number of animal tissues^13–15^.

Different tissues consist of different types of cells. Although many metabolic cycles are shared between cell types, the contributions of these cycles into the total metabolic activity are different. Metabolites play an important role in maintaining the cell homeostasis and cellular protection, the protection against oxidative and osmotic stresses in particular. Different cell types may contain different combinations of antioxidants and osmolytes. Thus, one can expect considerable difference between metabolomic compositions of tissues, and it would be of fundamental importance to determine the levels of major metabolites in different tissues of the same species. That will bring a better understanding of mechanisms of cellular protection and of roles of specific metabolic cycles in different tissues. In our recent work^15^, we compared the metabolomic compositions of lenses and gills of freshwater fish—pike-perch (*Sander lucioperca*) and Siberian roach (*Rutilus rutilus lacustris*). It was found that the sets of antioxidants and osmolytes in lenses and gills are different, and they undergo significant seasonal variations. We have also reported the discovery of very powerful natural antioxidant—ovothiol A (OSH) in the fish lens^12^. The origin of OSH in the lens remains uncertain, and it is interesting to monitor this antioxidant throughout the fish tissues.

This work is devoted to the quantitative metabolomic analysis of a large set of tissues belonging to the same species, *S. lucioperca*, including gill, heart, liver, kidney, spleen, muscle, brain, milt, lens, aqueous (AH) and vitreous (VH) humors. The major goal of the study is to establish the baseline metabolite concentrations in these tissues. The comparison of metabolomic compositions of different tissues will also help to determine the major osmolytes and antioxidants, and to evaluate the roles of metabolic processes specific for every particular tissue.

## Results

**Figure 1:**
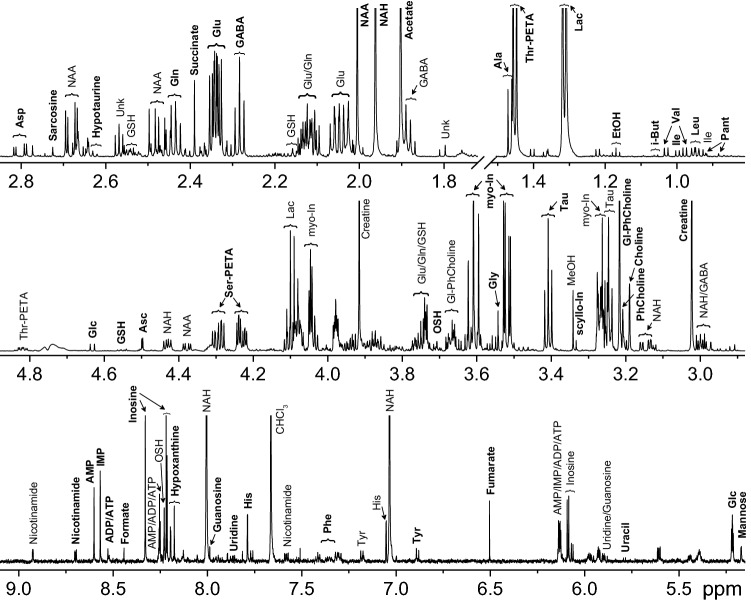
Representative ^1^H NMR spectra of protein-free lipid-free extract from *S. lucioperca* brain with the metabolite assignment: *Asc* Ascorbate, *GABA* gamma-Amino-butyrate, *Glc* Glucose, *Gl-PhCholine* Glycerophosphocholine, *i-But* Isobutyrate, *Lac* Lactate, *myo-In* *myo*-Inositol, *NAA* *N*-acetyl-Aspartate, *NAH* *N*-acetyl-Histidine, *OSH* Ovothiol A, *Pant* Pantothenate, *PhCholine* Phosphocholine, *scyllo-In* *scyllo*-Inositol, *Ser-PETA* Serine-phosphoethanolamine, *Tau* Taurine, *Thr-PETA* Threonine-phosphoethanolamine, *Unk* Unknown. For amino acids and nucleotides standard 3-letter symbols are used.

Figure 1 shows an example of the NMR spectrum of protein-free lipid-free extract from the fish brain. Similar spectra were obtained for other fish tissues: gill, heart, liver, kidney, spleen, muscle, milt, lens, aqueous and vitreous humors; raw data are available at the MetaboLights repository, study identifier MTBLS1763 (https://www.ebi.ac.uk/metabolights/MTBLS1763). The spectra of *S. lucioperca* lens and gill have recently been published^15^ by our group. For the majority of metabolites, the identification was performed according to their NMR spectra available in literature^2^ and in our in-house library^9–11,15,16^ without additional confirmation. In some cases, the signal assignment was unobvious; in these cases, the identification was confirmed by spiking the extract with commercial standard compounds.

**Figure 2:**
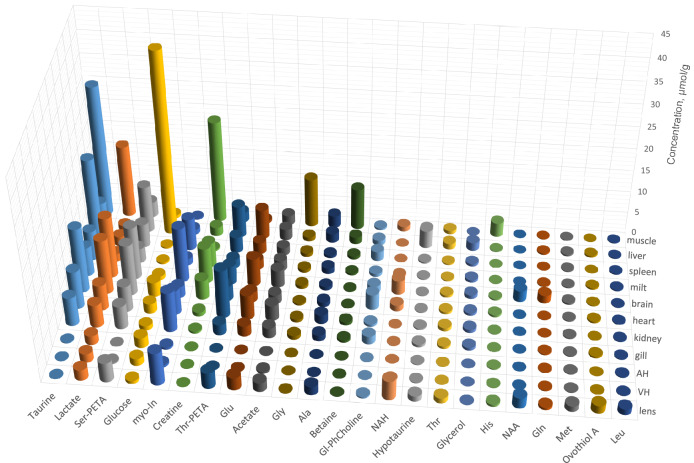
Graphical representation of mean concentrations of 23 major metabolites (in µmol/g) in *S. lucioperca* tissues.

The metabolite quantification was performed by the NMR signal integration relatively to the internal standard DSS followed by the calculation of metabolite concentrations in tissue (in nmoles per gram of the tissue wet weight). Typically, 65–70 compounds were identified in every sample; however, the NMR signals from some compounds were either too weak or strongly overlapped by other signals, which made the quantification of these compounds unreliable. For that reason, the final set of metabolites studied in this work was restricted to 66 compounds. For every tissue, the measurements were performed for 4–5 samples obtained from different species, the results were averaged. The obtained data are collected in Table 1 (mean ± std). Figure 2 demonstrates the graphical representation of the abundances of several major metabolites in the fish tissues.

**Table 1:** Concentrations (mean ± std, in nmol per gram of wet tissue) of metabolites in tissues of *Sander lucioperca.*

	AH	VH	Lens	Gill	Kidney	Milt	Heart	Spleen	Brain	Liver	Muscle
**Proteinogenic amino acids**											
Alanine	61 ± 7	74 ± 34	2000 ± 170	1300 ± 300	2000 ± 500	1300 ± 600	1000 ± 300	600 ± 210	770 ± 210	2100 ± 1100	2700 ± 1400
Aspartate	2.7 ± 0.8	11 ± 2	530 ± 30	200 ± 50	270 ± 130	370 ± 160	110 ± 40	260 ± 80	260 ± 80	97 ± 68	49 ± 19
Glutamate	27 ± 9	110 ± 60	3000 ± 300	2400 ± 300	5600 ± 400	2100 ± 600	2300 ± 500	2600 ± 800	6400 ± 500	6800 ± 3200	560 ± 110
Glutamine	120 ± 20	170 ± 40	550 ± 130	220 ± 40	53 ± 8	190 ± 50	110 ± 30	74 ± 31	1800 ± 100	300 ± 240	120 ± 50
Glycine	3.6 ± 1.2	20 ± 7	84 ± 23	800 ± 170	610 ± 180	660 ± 120	370 ± 50	770 ± 180	760 ± 190	900 ± 240	11,000 ± 1000
Histidine	190 ± 230	91 ± 47	460 ± 30	79 ± 16	100 ± 40	43 ± 9	420 ± 170	52 ± 22	290 ± 130	190 ± 70	3200 ± 1000
Isoleucine	65 ± 10	51 ± 15	410 ± 60	76 ± 15	120 ± 20	100 ± 4	80 ± 15	110 ± 30	62 ± 16	97 ± 19	110 ± 50
Leucine	190 ± 30	150 ± 30	1300 ± 200	170 ± 30	210 ± 70	230 ± 30	180 ± 40	240 ± 60	120 ± 20	190 ± 50	180 ± 80
Methionine	160 ± 50	140 ± 50	1300 ± 200	180 ± 10	200 ± 10	240 ± 40	200 ± 30	190 ± 50	380 ± 10	340 ± 140	170 ± 30
Phenylalanine	62 ± 2	54 ± 8	610 ± 90	72 ± 13	100 ± 30	79 ± 15	50 ± 9	100 ± 40	57 ± 11	31 ± 10	140 ± 60
Proline	ND^a^	ND	79 ± 31	160 ± 20	180 ± 70	110 ± 30	140 ± 70	NQ^b^	160 ± 60	NQ	340 ± 230
Serine	440 ± 100	280 ± 90	970 ± 130	570 ± 90	290 ± 70	540 ± 140	650 ± 140	400 ± 210	700 ± 100	NQ	480 ± 210
Threonine	68 ± 29	58 ± 27	1200 ± 300	510 ± 170	760 ± 250	190 ± 100	500 ± 210	260 ± 100	500 ± 200	1300 ± 500	740 ± 440
Tryptophan	23 ± 8	13 ± 8	390 ± 30	41 ± 17	22 ± 13	27 ± 8	20 ± 4	31 ± 17	14 ± 8	22 ± 4	40 ± 45
Tyrosine	72 ± 15	52 ± 21	970 ± 200	73 ± 7	82 ± 15	76 ± 4	47 ± 7	110 ± 40	46 ± 10	58 ± 15	52 ± 11
Valine	74 ± 8	59 ± 14	440 ± 90	130 ± 30	190 ± 50	160 ± 5	130 ± 29	150 ± 50	70 ± 16	140 ± 20	140 ± 50
**Other amino acids**											
Acetyl-Carnitine	1.9 ± 0.3	1.6 ± 0.4	27 ± 6	19 ± 5	7.0 ± 2.5	9.2 ± 7.7	58 ± 14	17 ± 14	15 ± 2	70 ± 47	83 ± 35
Carnitine	0.07 ± 0.23	2.9 ± 1.2	85 ± 8	140 ± 20	94 ± 33	93 ± 13	190 ± 60	210 ± 60	61 ± 7	140 ± 2	54 ± 21
Creatine	8.3 ± 1.1	87 ± 45	37 ± 8	420 ± 110	910 ± 260	5500 ± 2400	4500 ± 1200	380 ± 180	8100 ± 600	2000 ± 500	23,000 ± 1000
Hypotaurine	100 ± 30	92 ± 33	830 ± 130	390 ± 50	260 ± 70	120 ± 20	100 ± 20	230 ± 50	120 ± 20	2700 ± 1000	470 ± 40
Ketoleucine	4.3 ± 0.9	1.7 ± 0.8	14 ± 6	2.2 ± 1.4	1.9 ± 1.8	2.7 ± 1.2	2.2 ± 0.6	ND	2.7 ± 1.1	ND	2.8 ± 1.6
Ornithine	7.2 ± 1.2	7.9 ± 0.3	35 ± 12	62 ± 15	82 ± 33	36 ± 20	62 ± 27	34 ± 35	61 ± 7	110 ± 50	130 ± 90
Sarcosine	0.68 ± 0.61	1.9 ± 1.2	19 ± 9	17 ± 4	86 ± 28	26 ± 8	14 ± 6	30 ± 11	53 ± 12	74 ± 26	12 ± 2
**Organic acids**											
2-Hydroxy-butyrate	11 ± 4	7.7 ± 4.3	9.8 ± 4.1	5.2 ± 1.5	4.5 ± 1.4	ND	6.1 ± 2.4	ND	8.6 ± 2.3	ND	ND
3-Amino-isobutyrate	ND	ND	1.7 ± 1.9	22 ± 8	8.0 ± 4.9	4.7 ± 1.3	3.8 ± 4.3	5.1 ± 3.0	8.3 ± 1.6	31 ± 19	29 ± 10
Acetate	59 ± 5	380 ± 70	2100 ± 500	2300 ± 700	3500 ± 600	2400 ± 500	2000 ± 600	1700 ± 300	3900 ± 700	2500 ± 500	2100 ± 200
AABA	5.2 ± 1.7	2.9 ± 1.8	61 ± 26	15 ± 4	20 ± 21	21 ± 15	13 ± 6	8.9 ± 2.1	6.5 ± 1.6	45 ± 22	33 ± 11
Formate	230 ± 40	110 ± 50	17 ± 4	26 ± 7	37 ± 8	35 ± 8	20 ± 2	34 ± 4	19 ± 3	32 ± 8	24 ± 6
Fumarate	0.37 ± 0.38	2 ± 1.2	11 ± 3	56 ± 15	91 ± 50	15 ± 5	50 ± 14	18 ± 9	57 ± 16	160 ± 80	42 ± 7
GABA	ND	20 ± 11	6.5 ± 1.7	79 ± 16	56 ± 45	15 ± 17	8.7 ± 1.7	36 ± 16	1300 ± 300	40 ± 31	9.1 ± 4.4
Isobutyrate	15 ± 3	10 ± 3	5.3 ± 1.5	5.9 ± 2.3	5.4 ± 2.0	4.1 ± 0.3	4.6 ± 1.3	2.9 ± 0.5	8.9 ± 1.9	3.0 ± 1.4	0.9 ± 0.9
Lactate	2000 ± 500	2000 ± 600	2500 ± 400	5200 ± 1000	5900 ± 1000	1700 ± 700	1200 ± 400	2100 ± 700	14,000 ± 3000	1100 ± 200	16,000 ± 5000
Pantothenate	7.3 ± 2.9	5.2 ± 2.5	9.6 ± 3.2	14 ± 5	19 ± 8	ND	20 ± 5	10 ± 3	20 ± 5	15 ± 5	ND
Pyruvate	33 ± 8	7.9 ± 2.7	9.1 ± 3.0	11 ± 2	9.7 ± 2.1	15 ± 15	8.6 ± 1.4	20 ± 16	14 ± 4	19 ± 7	21 ± 18
Succinate	7.4 ± 0.2	12 ± 4	82 ± 9	83 ± 21	390 ± 150	85 ± 38	190 ± 110	94 ± 57	190 ± 30	810 ± 370	210 ± 60
**Osmolytes**											
Betaine	2.2 ± 1.4	5.7 ± 2.7	82 ± 18	530 ± 90	670 ± 170	340 ± 60	160 ± 50	700 ± 300	160 ± 30	1500 ± 500	9400 ± 1800
*myo*-Inositol	410 ± 30	480 ± 150	7700 ± 700	9200 ± 700	5600 ± 1700	720 ± 210	2500 ± 300	7300 ± 1700	13,000 ± 1000	1100 ± 100	250 ± 70
NAA	23 ± 5	50 ± 29	2400 ± 300	ND	11 ± 3.0	10 ± 3	22 ± 3	3.9 ± 2.1	2500 ± 300	9.8 ± 5.3	42 ± 18
NAH	6.8 ± 0.9	99 ± 71	4400 ± 800	68 ± 10	5.6 ± 1.1	450 ± 70	1300 ± 300	12 ± 3	3200 ± 300	29 ± 13	1100 ± 700
Ser-PETA	ND	250 ± 100	4500 ± 800	5300 ± 600	5900 ± 1100	2400 ± 800	12,000 ± 2000	5400 ± 2100	12,000 ± 2000	11,000 ± 2000	3500 ± 400
Taurine	140 ± 30	60 ± 16	100 ± 8	6600 ± 700	8700 ± 1100	4100 ± 900	15,000 ± 3000	20,000 ± 3000	4200 ± 300	6100 ± 3300	30,000 ± 3000
Thr-PETA	14 ± 6	94 ± 42	3700 ± 300	2400 ± 400	11,000 ± 3000	520 ± 0	3400 ± 700	3600 ± 1500	4200 ± 300	7300 ± 2200	2300 ± 200
**Antioxidants**											
Ascorbate	4.4 ± 1.0	8.3 ± 4.0	NQ	180 ± 40	93 ± 58	NQ	NQ	46 ± 12	430 ± 50	NQ	NQ
GSH	ND	ND	490 ± 90	NQ	5.8 ± 3.2	200 ± 70	290 ± 100	ND	180 ± 90	470 ± 50	140 ± 130
Ovothiol A	5.0 ± 1.7	31 ± 12	1700 ± 300	370 ± 70	56 ± 64	330 ± 130	340 ± 30	ND	150 ± 40	360 ± 100	67 ± 34
**Alcohols, amines, sugars**											
Choline	5.2 ± 0.7	20 ± 9	110 ± 30	2000 ± 400	1600 ± 570	810 ± 900	830 ± 200	2000 ± 700	730 ± 280	1700 ± 500	140 ± 30
Ethanolamine	35 ± 9	73 ± 30	190 ± 30	1000 ± 100	1100 ± 400	700 ± 350	880 ± 205	680 ± 310	750 ± 130	860 ± 270	ND
Glucose	2300 ± 600	1600 ± 400	640 ± 70	1000 ± 300	2200 ± 400	370 ± 320	3300 ± 600	260 ± 120	840 ± 150	42,000 ± 11,000	660 ± 440
Glycerol	5.8 ± 3.5	10 ± 5	48 ± 24	600 ± 100	720 ± 320	560 ± 380	580 ± 110	530 ± 250	840 ± 180	1800 ± 600	66 ± 42
Glycerophosphocholine	5.3 ± 0.6	30 ± 11	280 ± 40	1800 ± 200	570 ± 90	660 ± 210	3500 ± 1100	2100 ± 400	1600 ± 100	1100 ± 500	410 ± 60
Mannose	21 ± 14	18 ± 7	16 ± 9	51 ± 15	61 ± 5	25 ± 10	290 ± 120	22 ± 9	160 ± 50	480 ± 150	80 ± 66
Phosphocholine	3.8 ± 2.6	16 ± 7	1100 ± 100	620 ± 100	650 ± 160	430 ± 150	300 ± 120	1100 ± 300	360 ± 80	600 ± 190	95 ± 45
*scyllo*-Inositol	3.6 ± 0.2	5.1 ± 1.1	27 ± 4	170 ± 20	230 ± 100	26 ± 14	24 ± 3	58 ± 24	76 ± 17	39 ± 10	24 ± 11
**Nitrogenous bases, nucleotides, nucleosides**											
ADP	ND	ND	170 ± 34	50 ± 12	140 ± 90	110 ± 44	14 ± 3	250 ± 200	57 ± 17	12,930 ± 70	510 ± 80
AMP	ND	ND	41 ± 3	81 ± 41	270 ± 200	900 ± 420	110 ± 60	180 ± 150	200 ± 70	630 ± 190	60 ± 28
ATP	ND	ND	720 ± 100	23 ± 12	140 ± 100	31 ± 14	180 ± 440	230 ± 280	56 ± 20	140 ± 50	120 ± 50
Creatinine	4.2 ± 0.6	3.5 ± 1.0	49 ± 12	15 ± 5	4.8 ± 2.6	ND	11 ± 3	21 ± 14	38 ± 25	27 ± 13	82 ± 39
Guanosine	ND	6.9 ± 3.5	18 ± 27	89 ± 14	47 ± 9	15 ± 22	59 ± 29	230 ± 100	32 ± 8	56 ± 27	3.7 ± 2.7
Hypoxantine	ND	ND	43 ± 5	240 ± 60	600 ± 180	75 ± 34	35 ± 11	180 ± 60	130 ± 50	110 ± 80	29 ± 8
Inosinate	ND	ND	27 ± 4	86 ± 25	140 ± 120	160 ± 70	1100 ± 500	100 ± 160	200 ± 20	340 ± 150	450 ± 270
Inosine	ND	17 ± 15	18 ± 6	830 ± 70	920 ± 500	230 ± 250	650 ± 110	1500 ± 700	420 ± 140	580 ± 260	290 ± 170
NAD	ND	ND	94 ± 9	ND	17 ± 8	21 ± 13	23 ± 9	12 ± 7	ND	52 ± 26	25 ± 9
NADH	ND	ND	3.8 ± 2.6	ND	1.1 ± 1.8	ND	4.7 ± 2.3	ND	ND	10 ± 6	3.2 ± 1.1
Nicotinamide	ND	ND	ND	67 ± 6	180 ± 20	33 ± 26	170 ± 40	69 ± 20	83 ± 10	190 ± 40	28 ± 13
Uracil	ND	ND	ND	85 ± 17	59 ± 24	20 ± 21	12 ± 3	31 ± 22	17 ± 9	25 ± 12	9.6 ± 5.4
Uridine	1.6 ± 0.8	5.0 ± 2.7	6.7 ± 6.4	43 ± 8	65 ± 18	16 ± 28	26 ± 6	68 ± 29	33 ± 13	41 ± 10	3.8 ± 1.4

^a^*ND* not detected.

^b^*NQ* detected but not quantified.

**Figure 3:**
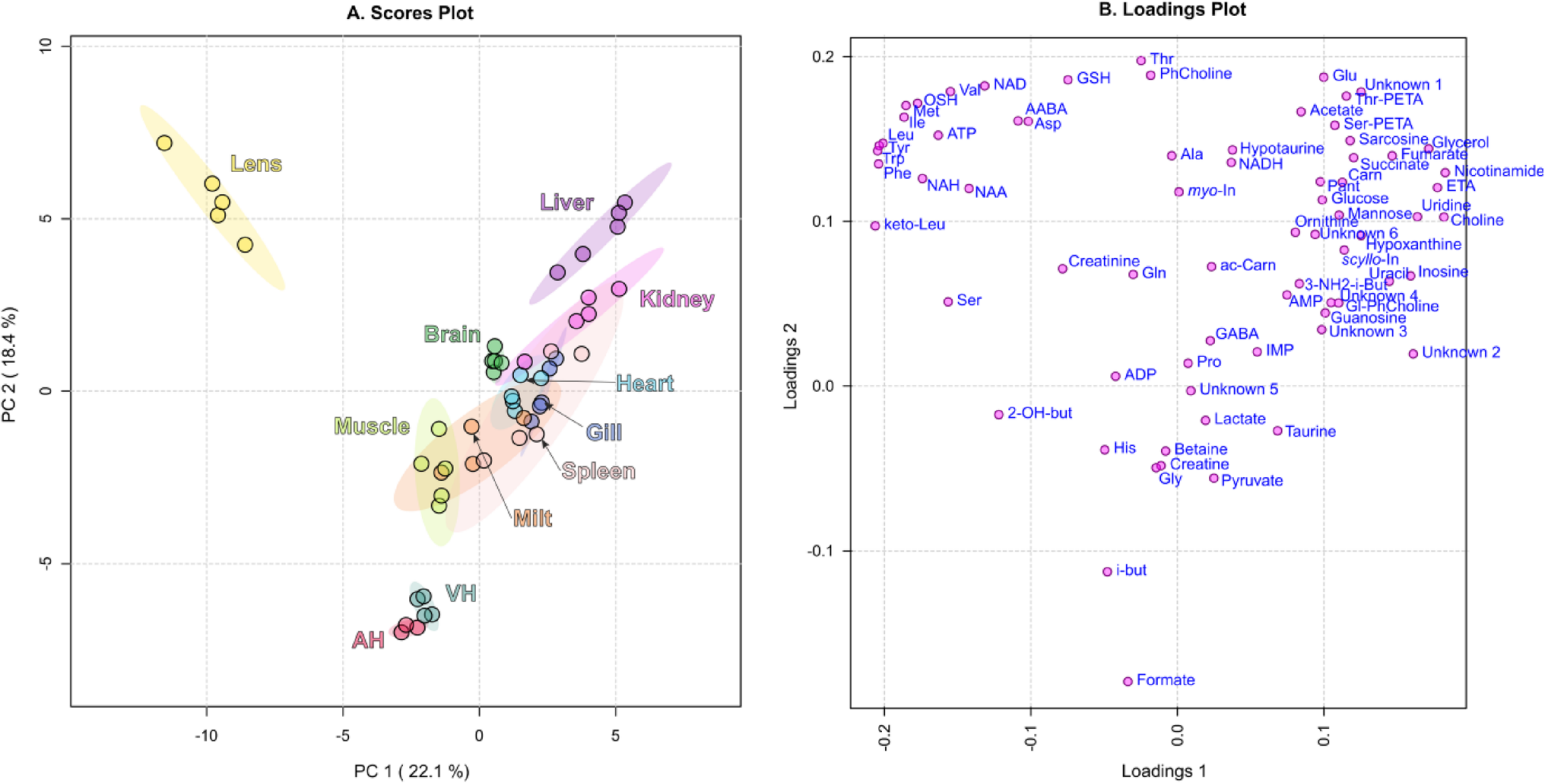
Scores (**A**, left) and loadings (**B**, right) plots of principal component analysis (PCA) of metabolomic profiles of *S. lucioperca* tissues. The data are auto scaled. Colored ovals indicate 95% confidence regions. Variance explained by the first (PC1) and second (PC2) principal components are indicated on the axis of scores plot.

Figure 3 shows the PCA plots for the 1st versus 2nd principal components built for all eleven tissues studied in the present work. It demonstrates that the lens metabolomic composition most drastically differs from metabolomes of other tissues: the 1st principal component is almost completely determined by the metabolomic difference between the lens and other tissues (provided mostly by amino acids, *N*-acetyl-histidine (NAH), *N*-acetyl-aspartate (NAA), nicotinamide adenine dinucleotide (NAD), adenosine triphosphate (ATP), and OSH; Fig. 3B). All other tissues in Fig. 3A vary mostly along the 2nd principal component with AH and VH being at the very bottom of the plot and liver—at the top. The data for muscle, heart, kidney, spleen, brain, gill, and milt are concentrated in the same region. Apparently, that does not mean that the metabolomic compositions of these tissues are almost identical; that rather indicates that the difference between the lens and these tissues is less pronounced than the difference between the lens and AH, VH, and liver.

**Figure 4:**
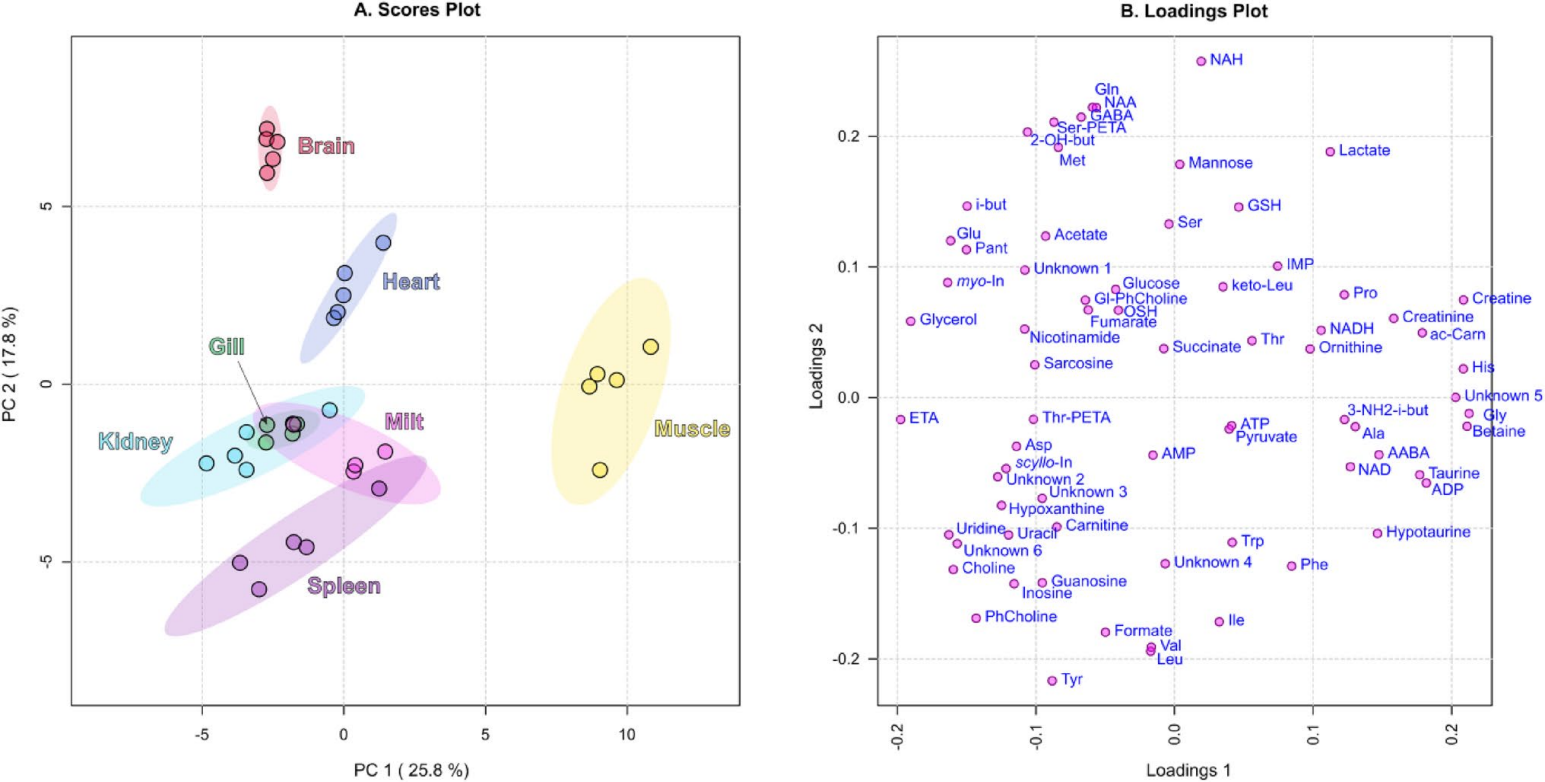
Scores (**A**, left) and loadings (**B**, right) plots of principal component analysis (PCA) of metabolomic profiles of selected *S. lucioperca* tissues (AH, VH, liver, and lens are excluded). The data are auto scaled. Colored ovals indicate 95% confidence regions. Variance explained by the first (PC1) and second (PC2) principal components are indicated on the axis of scores plot.

To compare the metabolomic profiles of muscle, heart, kidney, spleen, brain, gill, and milt, the PCA was performed for these tissues only (Fig. 4). The scores plot (Fig. 4A) shows that among these tissues, muscle and brain have the most distinct metabolomic compositions, while the profiles of gill, kidney, milt, and spleen are relatively similar. The loadings plot (Fig. 4B) demonstrates that the difference between brain and other tissues is mostly provided by the high abundance of NAH, NAA, glutamine, gamma-amino-butyrate (GABA), and serine-phosphoethanolamine (Ser-PETA), while specific for muscle metabolites are creatine, histidine, glycine, and betaine.

**Figure 5:**
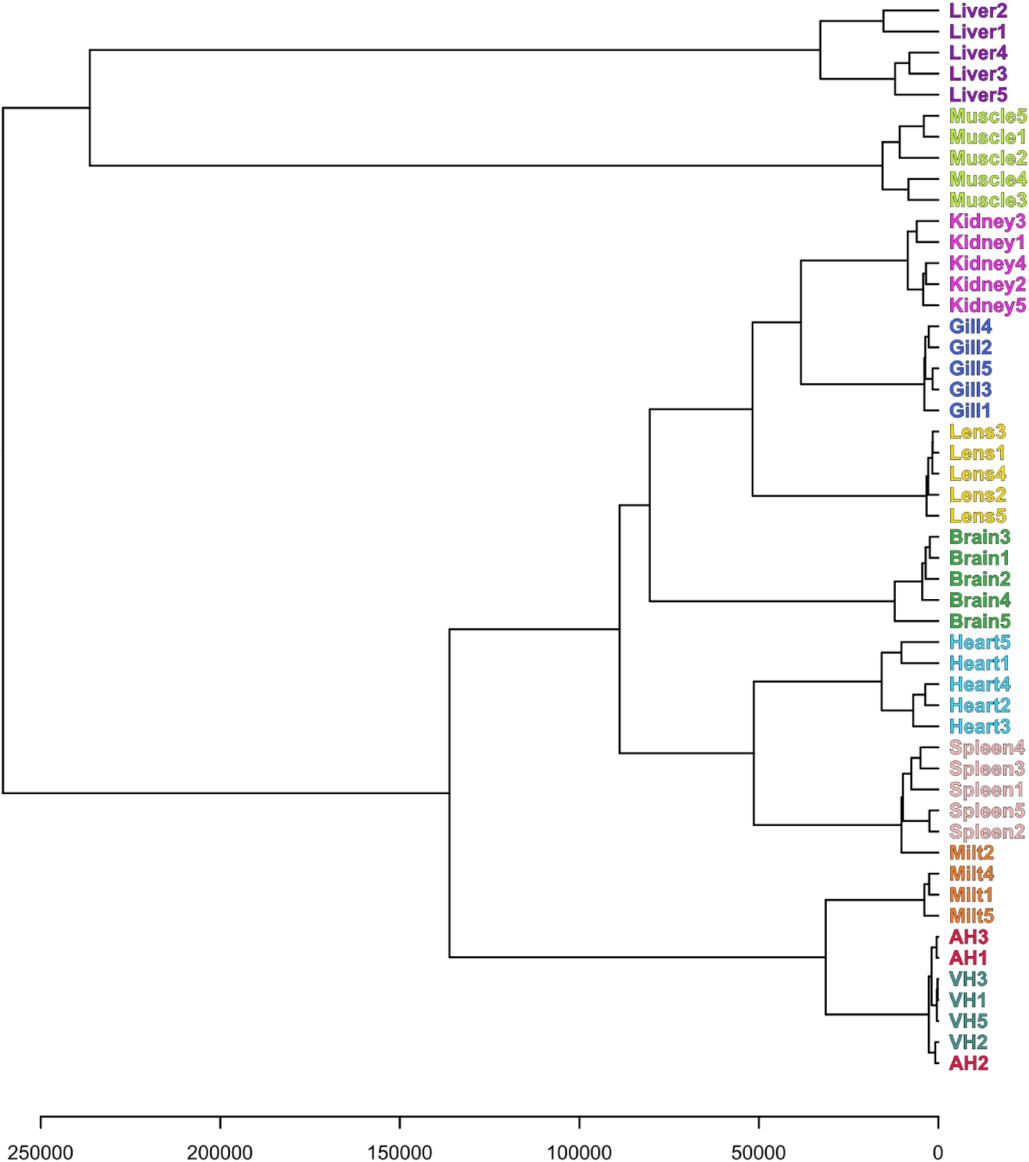
Hierarchical clustering analysis of metabolomic profiles of *S. lucioperca* tissues performed on non-scaled data with the use of Euclidian distance and Ward’s linkage.

The analysis of similarities of metabolomic compositions of different tissues can be performed with the use of hierarchical clustering (Fig. 5). Hierarchical clustering analysis (HCA) as opposed to PCA was performed for non-scaled data with the use of Euclidian distance and Ward’s linkage as the clustering parameters. HCA shows that all samples are grouped first of all by tissues indicating low dispersion of concentrations in each tissue and noticeable difference between tissues (with the exception of AH and VH). AH and VH have very similar metabolomic compositions: as it will be discussed below, the metabolomes of both fluids originate from the blood plasma metabolome. The metabolomic composition of milt is closest to that of AH and VH. In Fig. 5, one can observe the following groups of tissues with relatively similar metabolomes: kidney and gills; heart and spleen. That is in a good agreement with the PCA results (Figs. 3, 4). According to HCA, liver and muscle are characterized by the most unique and distinct metabolomic compositions.

## Discussion

In the present work, we performed quantitative metabolomic analysis for eleven biological tissues of *S. lucioperca*. The advantage of the quantitative approach over commonly used semi-quantitative measurements is that the obtained data on the metabolite concentrations expressed in nmoles per gram of a tissue can be directly used by any researcher as a reference to the baseline level of metabolites in that tissue. Quantitative data also allow for the comparing the tissues with very dissimilar metabolomic compositions.

The metabolomic analysis performed in the present work demonstrates that although the majority of metabolites are common for all tissues, their concentrations in tissues may vary at a large scale. Moreover, there are some tissue-specific compounds with very high abundance in only 1–2 types of tissue. The examples of such metabolites are glycine, histidine, creatine, and betaine in muscle, ovothiol A in lens, NAA in lens and brain, glucose in liver. Apparently, these compounds are important for biological functions specific for these particular tissues.

**Figure 6:**
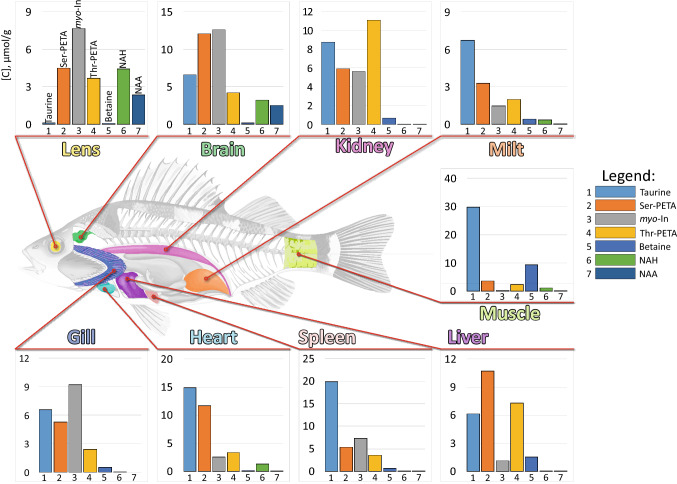
Concentrations of major osmolytes (in µmol/g) in *S. lucioperca* tissues.

Two groups of metabolites, osmolytes and antioxidants, play the key role in the cell protection against osmotic and oxidative stresses. In this work, the following compounds were conventionally assigned to osmolytes: taurine, *myo*-inositol, NAH, NAA, betaine, threonine-phosphoethanolamine (Thr-PETA), and Ser-PETA. Obviously, this assignment is rather arbitrary: some of these compounds, besides osmotic protection, perform other cellular functions, including cell signaling, providing substrate for biosynthesis, and so on^17–19^. At the same time, the tissues under study contain metabolites with concentrations of the same level or even higher than the concentrations of compounds assigned to osmolytes: lactate, glucose, acetate, creatine. These metabolites are mostly related to the reactions of cellular energy generation, and their concentrations should strongly depend on the fish activity. For that reason, in this work we did not include them into the list of osmolytes. Figure 6 shows the concentrations of osmolytes in different fish tissues (excluding acellular tissues AH and VH), and demonstrates that the composition of osmolytes in tissues strongly depends on the cell type.

We found in the fish tissues the following compounds with antioxidative properties: glutathione (GSH), ascorbate, OSH, and NADH. The detection of minor amounts of one more well-known thiol antioxidant, ergothioneine, in the gills of another freshwater fish, R. rutilus lacustris, has recently been reported^15^; however, in the present work ergothioneine was not found neither by NMR nor by LC–MS in any of the studied tissues of *S. lucioperca*. NADH was found only in NMR spectra of liver, muscle, heart, and lens, and its concentration in these tissues does not exceed 15 nmol/g. GSH, OSH, and ascorbate are present in much higher concentrations in the majority of the fish tissues, so these three compounds play the main role in the cellular defense against the oxidative stress. The presence and the relative abundance of GSH and OSH in the fish tissues were confirmed by LC–MS data.

The metabolomic features of particular fish tissues are discussed below.

### AH and VH

AH and VH are acellular fluids with minimal metabolic activity. AH is produced in the ciliary epithelium through both the active secretion and the passive diffusion/ultrafiltration of blood plasma^20–23^. Consequently, the metabolomic composition of AH is similar to that of plasma^10^. VH is also connected with blood via the hematoophthalmic barrier^24^ and with AH, and one can see (Table 1, Fig. 2) that the metabolomic compositions of AH and VH are close to each other. Thus, it is safe to assume that the levels of metabolites in AH and VH reflect their levels in blood plasma, which circulates through the majority of fish tissues. Significant deviations of metabolite levels in tissue as compared to plasma should be attributed to the intracellular metabolic activity specific for this particular tissue.

### Lens

The eye lens is one of the most anatomically isolated tissues. The lens mostly consists of metabolically inert fiber cells without nuclei and organelles with the exception of metabolically active epithelial monolayer. The data present in Table 1 indicate that the lens contains very high levels of proteinogenic amino acids: for some amino acids (for example, branched-chain amino acids, glutamine, aspartate) their levels in the lens are more than ten-fold higher than that in AH. Moreover, the concentrations of the majority of amino acids in the lens are higher than in any other fish tissue. The elevated levels of amino acids in the lens has been noticed many years ago^25^, and it was attributed to the active amino acid transport from AH to lens^26–29^. These amino acids are presumably needed to synthesize high protein content (up to 40% of the total lens weight), which is in turn needed to provide high refraction coefficient.

The fish lens contains a unique set of osmolytes and antioxidants. The lens osmolytes are *myo*-inositol, NAH, NAA, Thr-PETA, and Ser-PETA. We have previously shown^15^ that the concentrations of osmolytes in the fish lens undergo significant seasonal variations. At the late winter time, when the fish was caught for this study, the most abundant lens osmolyte is *myo*-inositol. High concentrations of this compound are also found in other fish tissues, including brain, gill, and spleen. Thr-PETA and Ser-PETA are also among the most abundant metabolites in the majority of the fish tissues. In opposite, NAH and NAA are present in high concentrations only in the fish lens and brain. At the same time, the concentration of taurine, which is the most abundant osmolyte in all other fish tissues, in the lens is rather low.

The major antioxidant of the fish lens is OSH^12^. It has been shown that the level of OSH in *S. lucioperca* lens vary from 3 µmol/g at autumn to 1.5 µmol/g at winter^15^, which is in a good agreement with our present data (Table 1). The concentration of the second most abundant lens antioxidant, GSH, is 3–4 times lower than that of OSH. Taking into account the properties of OSH^30–33^, it has been proposed^12,34^ that OSH is a primary protector against the oxidative stress, while the main function of GSH in the lens is the maintenance of OSH in the reduced state. It should be noticed that although OSH was also found in other fish tissues (Table 1, Fig. 2), its concentration in these tissues is significantly lower than in the lens. Therefore, in respect to fish, OSH can truly be called “lenticular antioxidant”.

**Figure 7:**
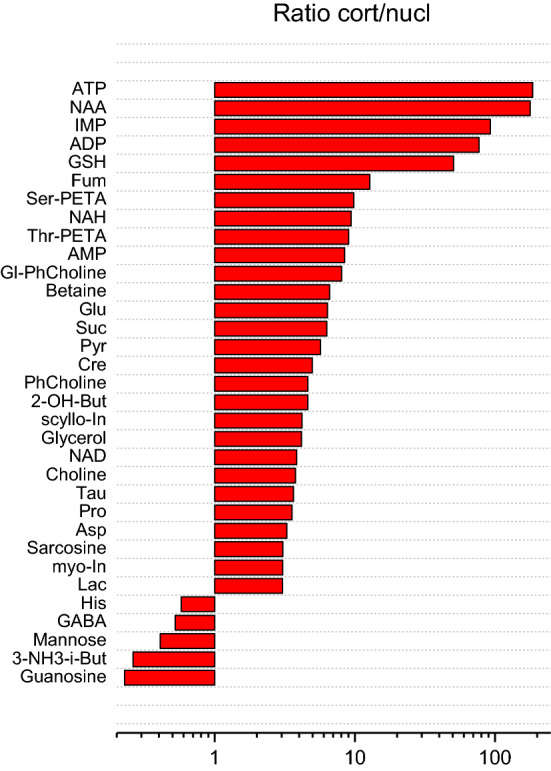
Barplot for statistically significant differences in the metabolomic content of lens cortex and nucleus. Bars show the averaged ratio of metabolite concentrations in the cortex to that in the nucleus of the *S. lucioperca* lens.

High concentrations of amino acids, osmolytes, antioxidants and some other compounds in the lens indicate that these metabolites are either synthesized in metabolically active epithelial cells, or pumped into the lens from AH against the concentration gradient with the use of specific transporters also located in the epithelial layer. The fiber cells of the lens are metabolically passive, and fresh metabolites can appear in these cells only due to the diffusion from the epithelial layer toward the lens center. Therefore, one can expect that the concentrations of the most important metabolites decrease from the lens cortex toward the lens nucleus. To check this assumption, we measured the metabolomic profiles for cortex and nucleus separately. The measurements were performed for three lenses; then the ratios of the metabolite concentrations in the cortex to that in the nucleus were calculated and averaged. The results of the calculations are shown in Fig. 7 (only for metabolites with the highest and the lowest cortex/nucleus ratios) and Supplementary Table S2 (for all metabolites). Indeed, the levels of the majority of metabolites in the lens nucleus are significantly lower than in the cortex. For five metabolites, namely ATP, NAA, inosinate, ADP, and GSH the difference exceeds the factor of thirty; that means that these compounds are almost completely depleted during their diffusion toward the lens nucleus.

### Brain

The brain tissue similarly to the lens is isolated from the vascular system by means of the hematoencephalic barrier. However, in opposite to the lens, brain is very metabolically active tissue, as in particular indicated by the high level of lactate (14 µmol/g). Similar lactate concentrations were found only in muscle and heart (Table 1). Besides lactate, the most abundant metabolites of the fish brain are osmolytes *myo*-inositol, Ser-PETA, taurine, Thr-PETA, NAH, and NAA; the concentrations of these compounds in brain are in the range from 2.5 to 13 µmol/g (Table 1). The brain tissue also contains high levels of glutamate and creatine, which are used by brain cells for the cellular energy generation. The level of antioxidant ascorbate in brain (400 nmol/g) is significantly higher than in other fish tissues, which indicates the importance of ascorbate for the brain correct operation. Besides ascorbate, the brain tissue also contains OSH and GSH, but at significantly lower concentrations (100–200 nmol/g).

### Blood-rich organs: liver, spleen, milt, muscle, heart, gill, kidney

Figure 4 demonstrates that from the metabolomic viewpoint, gill, kidney, milt, and spleen are the most similar tissues. However, the quantitative analysis indicates significant differences. In particular, spleen does not contain measurable by NMR amounts of antioxidants OSH and GSH. The levels of osmolytes are also different: the concentration of taurine in spleen is threefold higher than in gill and milt, while the level of *myo*-inositol in milt is much lower than in spleen and gill (Fig. 6). Significant differences are also found for some amino acids (alanine, creatine), organic acids (lactate, GABA), and nucleosides (ATP, ADP, AMP, inosine).

One of the important liver functions is the maintaining the glucose level in blood regulated by producing glucose from stored glycogen. Correspondingly, the level of glucose in liver is extremely high (40 µmol/g), which is higher than in any other tissue by at least an order of magnitude. Liver also contains elevated (as compared to other tissues) concentrations of threonine, glutamate, succinate, fumarate, AMP, and nicotinamide.

The biological functions of muscle and heart are relatively similar; however, the metabolomic compositions of these tissues differ significantly. The main osmolyte in muscle cells is taurine (30 µmol/g), while in heart the osmotic protection is shared between taurine (15 µmol/g) and Ser-PETA (12 µmol/g). Muscle contains very high levels of glycine and histidine. Glycine is known to protect muscles from wasting under various wasting conditions^35,36^, while histidine and histidine-related compounds were reported to play the role of intracellular proton buffering constituents in vertebrate muscle^37^. Very likely that both glycine and histidine also participate in the osmotic protection of the muscle cells. The level of creatine—the energy source—in muscle (23 µmol/g) is five-fold higher than in heart.

## Materials and methods

### Chemicals

Chloroform and methanol HPLC grade were purchased from Panreac (Spain). D_2_O 99.9% was purchased from Armar Chemicals (Switzerland). All other chemicals were purchased from Sigma-Aldrich (USA). H_2_O was deionized using an Ultra Clear UV plus TM water system (SG water, Germany) to the quality of 18.2 MOhm.

### Fish sample collection

The study was conducted in accordance with the ARVO Statement for the Use of Animals in Ophthalmic and Vision Research and the European Union Directive 2010/63/EU on the protection of animals used for scientific purposes, and with the ethical approval from International Tomography Center SB RAS. No special permission from the national or local authorities is required. *S. lucioperca* (males, body weight 800–1500 g) were caught in the ice-covered Ob reservoir with the use of a winter fishing rod at the beginning of April 2019 (n = 5). The fish were killed with a concussive blow to the head immediately after the catching, the tissues were cut from the fish, frozen and kept at − 70 °C until analyzed.

### Fish tissue preparation

The sample preparation was performed as described in Ref.^15^. Each fish tissue was weighted prior to homogenization. The typical sample weight was: for lens—230 mg; for heart—240 mg; for spleen—230 mg; for liver—200 mg; for muscle—200 mg; for brain—140 mg; for kidney—130 mg; for milt—170 mg; for gill—210 mg. The typical sample volumes were 300 μl for AH and 250 μl for VH. Only one lens from each fish was used for the analysis. The fish gill was divided into arch and filaments, only gill filaments were used for the analysis. Muscle tissue was excised from the area under the anal fin. Heart tissues were not subjected to the blood washing procedure.

Each fish tissue (except AH) was placed in a glass vial and homogenized with a TissueRuptor II homogenizer (Qiagen, Netherlands) in 1600 µL of cold (− 20 °C) MeOH, and then 800 µL of water and 1600 µL of cold chloroform were added^15^. The mixture was shaken well in a shaker for 20 min and left at − 20 °C for 30 min. Then the mixture was centrifuged at 16,100*g*, + 4 °C for 30 min, yielding two immiscible liquid layers separated by a protein layer. The upper aqueous layer (MeOH–H_2_O) was collected, divided into two parts for NMR (2/3) and LC–MS (1/3) analyses, and lyophilized. Since AH contains very low levels of proteins and lipids, it was extracted with MeOH/H_2_O solvent (1600/800 µL) without chloroform. The obtained protein-free and lipid-free extracts were used for NMR and LC–MS measurements.

To compare the metabolomic composition of the lens cortex and nucleus, the nucleus was separated from the cortex by coring the lens with a 3-mm home-made stainless steel borer followed by cutting off of approximately 1 mm from each end of the core^9^. The procedure was performed with lenses taken from storage at − 70 °C and warmed up to − 18 °C for easier cutting, all tools were cooled down to − 18 °C, and all manipulations were performed in a cold room at − 5 °C. The nucleus and the cortex (the combined doughnut-shaped outer remainder of boring and the ends of the core) were weighed and then extracted in the same way as other tissues.

### NMR measurements

The extracts for NMR measurements were re-dissolved in 600 μL of D_2_O containing 6 × 10^–6^ M sodium 4,4-dimethyl-4-silapentane-1-sulfonic acid (DSS) as an internal standard and 20 mM deuterated phosphate buffer to maintain pH 7.2. The ^1^H NMR measurements were carried out at the Center of Collective Use “Mass spectrometric investigations” SB RAS on a NMR spectrometer AVANCE III HD 700 MHz (Bruker BioSpin, Germany) equipped with a 16.44 T Ascend cryomagnet as described in Ref.^15^. The concentrations of metabolites in the samples were determined by the peak area integration respectively to the internal standard DSS.

### LC–MS measurements

In this work, LC–MS data were used only for the confirmation of data obtained by NMR method. The extracts for LC–MS analysis were re-dissolved in 100 μL of water. The LC separation was performed on an UltiMate 3000RS chromatograph (Dionex, Germering, Germany) using a hydrophilic interaction liquid chromatography (HILIC) method on a TSKgel Amide-80 h (Tosoh Bioscience, Griesheim, Germany) column (4.6 × 250 mm, 5 μm) as described earlier^16^. The chromatograph was equipped with a flow cell diode array UV–Vis detector (DAD) with 190–800 nm spectral range. Solvent A consisted of 0.1% formic acid solution in H_2_O, solvent B consisted of 0.1% formic acid solution in acetonitrile. The gradient was (solvent B): 95% (0–5 min), 95–65% (5–32 min), 65–35% (32–40 min), 35% (40–48 min), 35–95% (48–50 min), 95% (50–60 min); the flow rate was 1 mL/min, the sample injection volume was 10 μL. After the DAD cell, a home-made flow splitter (1:10) directed the lesser flow to an ESI-q-TOF high-resolution hybrid mass spectrometer maXis 4G (Bruker Daltonics, Bremen, Germany). The mass spectra were recorded in a positive mode with 50–1000 *m/z* range.

### Data analysis

To explore the data and to display the general metabolomic features in the data, the principal component analysis (PCA) has been performed on a MetaboAnalyst 4.0 web-platform (www.metaboanalyst.ca^38^). PCA scores and loadings plots were constructed with the auto data scaling (mean-centered and divided by standard deviation of each metabolite concentration) to normalize the contributions of all metabolites.

## Conclusions

The results of the present work demonstrate that NMR spectroscopy is an extremely useful tool for quantitative metabolomics; it allows for the determining the actual concentrations of up to hundred metabolites in biological fluids and tissues. The quantitative data indicate the most abundant compounds in every particular tissue, and, correspondingly, point to the most important and active metabolic processes in the tissue. It is important to notice that the metabolomic composition of tissues of fish and other marine and freshwater animals might be affected by a number of factors, including water temperature^39,40^, oxygen level^15,41^, and water contamination^42–45^. Therefore, the obtained quantitative data on the metabolite concentrations in the fish tissues correspond to rather specific conditions: species—*Sander lucioperca*, season—late winter, water temperature 4–7 °C, low level of dissolved oxygen in the ice-covered lake, moderate water pollution with the city waste. These data can be used as the baseline levels of metabolites for the analysis of peculiarities of metabolic processes in different species, and for the evaluation of influence of seasonal, ecological and other factors on the fish metabolism.

## Supplementary information

Supplementary Information.

## Data Availability

The data obtained in this study including NMR raw data, metabolite concentrations and experimental protocols have been deposited in MetaboLights repository, study identifier MTBLS1057 (https://www.ebi.ac.uk/metabolights/MTBLS1057).
